# Comparison of the protective effects of CS/TPP and CS/HPMCP nanoparticles containing berberine in ethanol-induced hepatotoxicity in rat

**DOI:** 10.1186/s12906-023-04318-9

**Published:** 2024-01-15

**Authors:** Maral Mahboubi Kancha, Morteza Alizadeh, Mohsen Mehrabi

**Affiliations:** 1 Student Research Committee, School of Medicine, Shahroud University of Medical Sciences, Shahroud, Iran; 2 Department of Tissue Engineering, School of Medicine, Shahroud University of Medical Sciences, Shahroud, Iran; 3 Department of Medical Nanotechnology, School of Medicine, Shahroud University of Medical Sciences, Shahroud, Iran

**Keywords:** Chitosan, Nanoparticle, Hepatotoxicity, Berberine, HPMCP, TPP

## Abstract

**Background:**

Alcoholic liver disease (ALD) is a globally critical condition with no available efficient treatments.

**Methods:**

Herein, we generated chitosan (CS) nanoparticles cross-linked with two different agents, hydroxypropyl methylcellulose phthalate (HPMCP; termed as CS/HPMCP) and tripolyphosphate (TPP; termed as CS/TPP), and loaded them with berberine (BBr; referred to as CS/HPMCP/BBr and CS/TPP/BBr, respectively). Alongside the encapsulation efficiency (EE) and loading capacity (LC), the releasing activity of the nanoparticles was also measured in stimulated gastric fluid (SGF) and stimulated intestinal fluid (SIF) conditions. The effects of the prepared nanoparticles on the viability of mesenchymal stem cells (MSCs) were also evaluated. Ultimately, the protective effects of the nanoparticles were investigated in ALD mouse models.

**Results:**

SEM images demonstrated that CS/HPMCP and CS/TPP nanoparticles had an average size of 235.5 ± 42 and 172 ± 21 nm, respectively. The LC and EE for CS/HPMCP/BBr were calculated as 79.78% and 75.79%, respectively; while the LC and EE for CS/TPP/BBr were 84.26% and 80.05%, respectively. pH was a determining factor for releasing BBr from CS/HPMCP nanoparticles as a higher cargo-releasing rate was observed in a less acidic environment. Both the BBr-loaded nanoparticles increased the viability of MSCs in comparison with their BBr-free counterparts. In vivo results demonstrated CS/HPMCP/BBr and CS/TPP/BBr nanoparticles protected enzymatic liver functionality against ethanol-induced damage. They also prevented histopathological ethanol-induced damage.

**Conclusions:**

Crosslinking CS nanoparticles with HPMCP can mediate controlled drug release in the intestine improving the bioavailability of BBr.

## Background

Alcoholic liver disease (ALD) is one of the most common liver-related conditions in numerous countries [1–3]. It includes excessive alcohol consumption exceeding a particular daily amount and has various manifestations such as chronic hepatitis with liver fibrosis or cirrhosis [2]. ALD clinically encompasses a range of liver-related conditions starting with steatosis, which can advance to fibrosis and consequently result in cirrhosis in one out of every four patients who are frequent alcohol consumers [2]. According to the statistics, ALD-related mortality is more prevalent among men in the US. However, it has been demonstrated that ALD-related mortality in women occurs two to three years earlier than in men [1]. To this date, the underlying mechanism of ALD has not been fully elucidated. However, it has been found that prolonged excessive alcohol consumption can result in the formation of oxidative stress through amplified metabolism via the cytochrome P450 2E1 system [3]. This phenomenon can lead to the production of reactive oxygen species (ROS) and protein and DNA adducts. These products induce inflammatory signaling pathways within the liver, resulting in the expression of pro-inflammatory mediators. These pro-inflammatory mediators can mediate apoptosis and necrosis of hepatocytes [3]. Moreover, intra-hepatocyte mitochondrial stress resulting from ROS exposure can also cause structural and functional mitochondrial complications leading to an increased incidence of apoptosis. Additionally, epigenetic regulation has also been reported to be directly affected by alcohol consumption. Elevated levels of histone acetylation and methylation and particular site-specific histone acetylation can impede various antioxidant pathways and reactions and induce the expression of important pro-inflammatory genes [3].

Early-stage ALD usually does not have any physical manifestations. ALD is commonly diagnosed when it has reached an advanced stage with apparent manifestations. However, routine medical examinations can be highly beneficial for the early diagnosis of ALD. Histologic tests as well as evaluation of the level of liver enzymes are used as the common diagnostic methods [4]. The most important step for ALD treatment is retraining from further alcohol consumption. Moreover, liver transplantation is currently the only long-term management option available for individuals with decompensated liver cirrhosis [5]. Several types of medications, including corticosteroids, are also used under certain conditions [6]. Additionally, researchers have recently focused on monoclonal antibody therapeutics such as infliximab; however, the results regarding their clinical benefit are still unclear [7].

Berberine (BBr) is an alkaloid utilized as an herbal medicine for treating various types of health-related conditions, such as diarrhea, in traditional Chinese medicine [8]. Recently, various studies have highlighted the beneficial biological effects of berberine, which include antitumor effects, cardiovascular-protective properties, and anti-inflammatory activities [9]. To this date, many bodies of research have focused on the protective effects of BBr in various types of hepatotoxicity at different experimental levels. For instance, Germoush et al. investigated the protective properties of BBr in alleviating cyclophosphamide-induced hepatotoxicity in mouse preclinical models [10]. According to their results, oral administration of BBr for days after the administration of a single dose of cyclophosphamide improved the serum level of various hepatic enzymes which had deviated from the normal level following the administration of cyclophosphamide [10]. These researchers also indicated that BBr demonstrates noticeable hepatoprotective behavior against drug-induced hepatotoxicity [10]. Additionally, an investigation by Knittel et al. also demonstrated that oral administration of BBr with a dose of 25 and 50 mg/kg for 7 days can mediate hepatoprotective effects against methotrexate-induced liver toxicity [11]. Moreover, Wang et al. investigated the protective effects of BBr on liver fibrosis in rat models of liver fibrosis established using bile duct ligation (BDL) [12]. These researchers demonstrated that BBr prevents hepatic fibrosis mediated by BDL in preclinical mouse models [12]. However, it was also indicated that the antifibrotic properties of BBr in patients demand further investigations [12]. In 2020, Li et al. studied the action mechanism by which BBr exerts its therapeutic effects on ALD linked to the gut microbiota-immune system axis [13]. These researchers established preclinical animal models of ALD, assessed various biochemical factors, and performed histological evaluations [13]. According to their results, they first reported the favorable therapeutic effects of BBr on “acute-on-chronic” alcoholic hepatic damage [13]. Furthermore, these researchers focused on the action mechanism related to the gut microbiota-immune system axis [13]. It was elucidated that BBr activates a group of immune cells called granulocytic-myeloid-derived suppressor cell (G-MDSC)-like cells and increases the population of these cells in both the liver and blood [13]. Furthermore, these cells improved the hepatic damage mediated by alcohol in the liver of the studied animal models [13]. In addition, it was reported that BBr decreased the population of cytotoxic T cells [13]. Of note, these researchers added that inhibiting the G-MDSC-like cell population remarkably debilitated the protective activity of BBr against alcohol [13]. Additionally, the findings of Zhang et al. demonstrated that BBr can reduce alcohol-induced oxidative stress by decreasing hepatic lipid peroxidation, glutathione exhaust, and mitochondria oxidative damage in mouse models [14]. In vivo assessments have highlighted the role of BBr in preventing ethanol-mediated oxidative stress and macro steatosis. Briefly, BBr can inhibit the total cytochrome P450 2E1 or the mitochondria cytochrome P450 2E1 activity. It has also been demonstrated that BBr can reduce excessive alcohol consumption-induced lipid accumulation in the liver. Such findings can propose the capability of BBr to serve as a possible agent for preventing or managing ALD [14]. However, in addition to these preclinical studies, clinical trials are crucially required for investigating the therapeutic and hepatoprotective effects of BBr in liver-related conditions.

To this date, many researchers have used nanoparticles for the nanomedicine-based delivery of BBr for different aims based on their properties including cancer therapy and antibacterial applications [15–18]. Studies have focused on enhancing various properties of BBr by loading it onto nanomedicine delivery platforms since the promising potentials of BBr are mostly tackled by its poor level of aqueous solubility, strong hydrophobicity, low rate of absorption in the gastrointestinal, and rapid metabolism in the body [16, 19–22]. Nanomedicine-based delivery systems aim to alleviate various properties of BBr to further support its applications. For instance, Wang et al. have reported that the antitumor activity of BBr remarkably increases when encapsulated in solid lipid nanoparticles [16]. Moreover, other researchers demonstrated that the bioavailability of BBr can be improved when loaded in chitosan (CS) nanoparticles and these nanoparticles could exhibit enhanced anticancer activity against nasopharyngeal carcinoma cells [23]. Additionally, other researchers investigated BBr-loaded CS nanoparticles in scopolamine-induced Alzheimer’s-like disease preclinical rat models [24]. The results of this study demonstrated that CS nanoparticles enhanced the bioavailability, absorption, and brain drug uptake of BBr in rat models [24]. In addition, an in vitro experiment demonstrated that loading BBr in O-hexadecyl-dextran nanoparticles improved its cytoprotective properties and these nanoparticles can decrease the level of oxidative stress in hepatocytes of rats at a concentration 20 times lower than free BBr and prevent high glucose stress [25]. Such studies suggest that nanotechnology-based platforms might improve the physical, chemical, and biological behavior of BBr and support its further applications.

Different types of nanoparticles have been investigated for their applicability in ameliorating hepatoxicity. For instance, Bhattacharjee et al. investigated the protective characteristics of selenium nanoparticles against hepatotoxicity and genotoxicity induced by cyclophosphamide in preclinical mouse models [26]. These researchers reported that intraperitoneal administration of cyclophosphamide was used for the establishment of the models and selenium nanoparticles were given by oral gavages. According to the results of this report, the delivery of nanoparticles decreased the level of various hepatotoxicity factors including malonaldehyde (MDA), ROS, glutathione, and various antioxidant enzymes [26]. It also resulted in a reduced level of chromosomal abnormalities and DNA damage in bone marrow and lymphocytes [26]. Additionally, the protective effects of selenium nanoparticles were also observed in histopathological samples in preclinical models of hepatoxicity [26]. In another study by Tabbasam et al., the researchers investigated the protective effects of inorganic nanoparticle complexes against carbon tetrachloride-induced hepatotoxicity in preclinical mouse models [27]. In detail, these researchers generated three different types of nanoparticles (gold, silver, and zinc oxide) all loaded with doxorubicin. According to the results, nanoparticle-assisted delivery of doxorubicin resulted in a reduced level of liver fibrosis. Moreover, they reported that silver nanoparticles loaded with doxorubicin outperformed the other types of nanoparticles by mediating the level of hepatic enzymes closest to the control group [27]. Overall, it was reported that drug-loaded silver nanoparticles demonstrated remarkable protective effects against carbon tetrachloride-induced hepatotoxicity [27]. Based on these findings, we proposed that nanoparticles may be beneficial for the treatment of hepatoxicity. However, it is worth mentioning that there are also many bodies of research reporting the induction of hepatoxicity by nanoparticles as well [28–30]. For instance, it has been reported that silver nanoparticles can mediate the emergence of hepatotoxicity in male rats [29]. In this regard, the researchers have suggested that *Beta vulgaris* (beetroot) water extract as a potential therapeutic intervention following the administration of sliver nanoparticles for minimizing nanoparticle-induced hepatotoxicity [29]. Other researchers also demonstrated that the intraperitoneal administration of gold nanoparticles induces liver damage, and produces oxidative stress and fatty acid peroxidation [28]. It was also demonstrated that melanin exhibits advantageous characteristics in reducing the liver toxicities induced by gold nanoparticles [28]. Such data suggest that even though nanoparticles can be used for alleviating liver toxicities, they can be also a source for various types of liver toxicities which should be broadly investigated.

CS-based nanoparticles have been broadly investigated as ideal delivery vehicles in many studies, owing to their capability for improving BBr bioavailability [23, 31]. CS has chemical functional groups which can be efficiently modified for particular purposes [32]. Such properties render CS a polymer with a remarkable variety of possible functions. However, the gastrointestinal delivery of various types of cargo using nanoparticles is challenging since a pH gradient exists in the gastrointestinal tract [33]. Therefore, different CS-based formulations that are responsive to specific pH conditions can be useful in this regard. CS nanoparticles formulated by ionic cross-linking with hydroxypropyl methylcellulose phthalate (HPMCP) have been investigated as pH-responsive nanoparticles for the targeted delivery of various types of cargo and it has been demonstrated that they can be safe and efficient in terms of targeted drug delivery [34]. Herein, we generated CS nanoparticles and cross-linked them with tripolyphosphate (TPP; hereafter referred to as CS/TPP) or HPMCP (hereafter referred to as CS/HPMCP) and loaded these nanoparticles with BBr (hereafter referred to as CS/TPP/BBr and CS/HPMCP/BBr, respectively) and assessed different aspects of these nanoparticles in vitro and in vivo.

## Materials and methods

### Materials

**Fig. 1:**
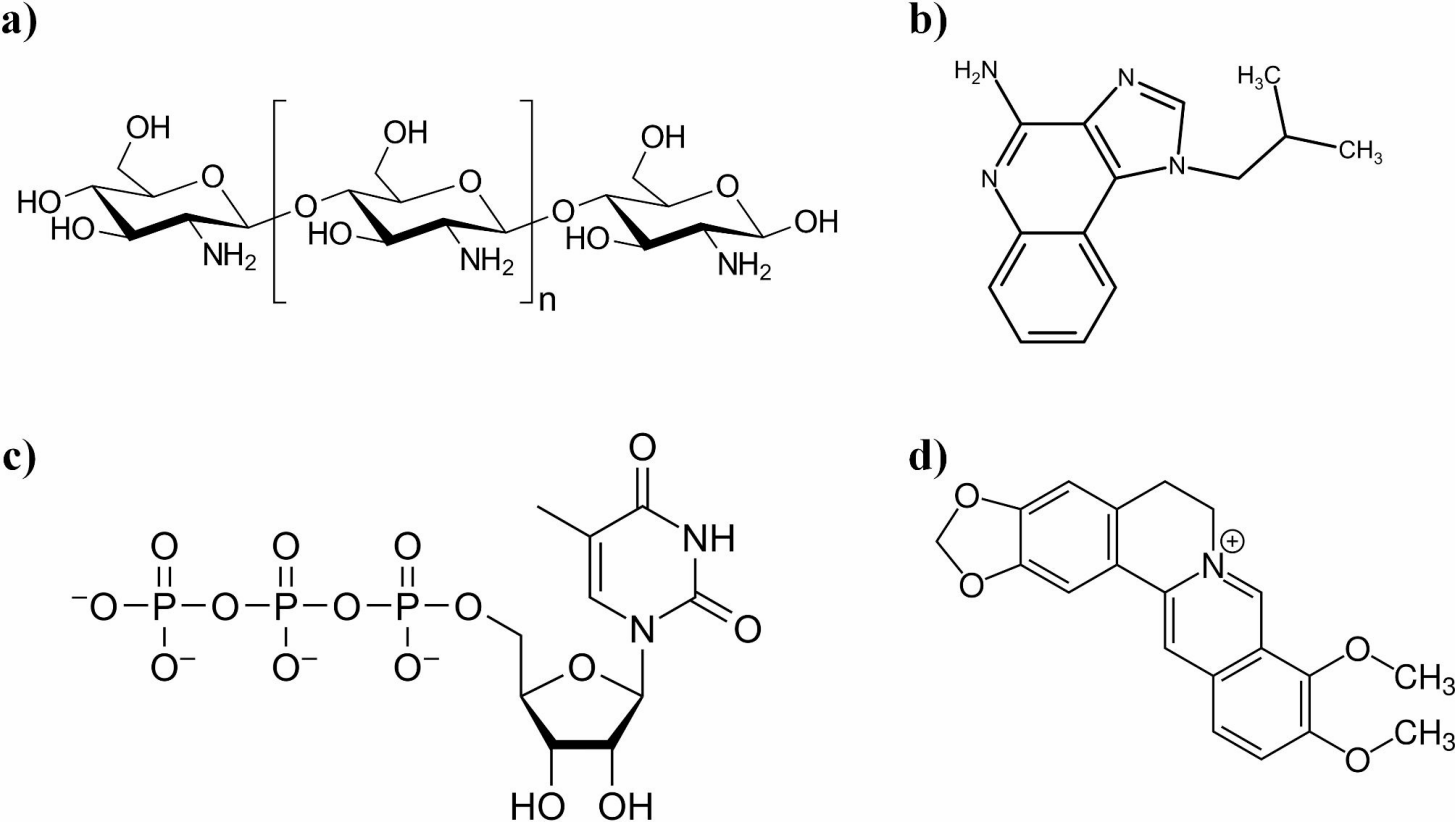
The chemical structures of CS, HPMCP, TPP, and BBr used in the preparation of nanoparticles that were investigated in this study. **a**: CS. **b**: HPMCP. **c**: TPP. **d**: BBr.

Low molecular weight CS was purchased from Primex Pharmaceuticals AG (Iceland). BBr chloride hydrate (catalog No. 14,050), pancreatin from porcine pancreas (catalog No. P3292), pepsin from porcine gastric mucosa (catalog No. P7000), dipotassium phosphate, diethyl ether, TPP, HPMCP, and 3-(4, 5-dimethylthiazol-2-yl)-2,5-diphenyltetrazolium bromide (MTT) were purchased from Sigma-Aldrich (USA). Penicillin-streptomycin, phosphate buffer saline (PBS), trypsin 0.25%, dimethyl sulfoxide (DMSO), sodium hydroxide (NaOH), heat-inactivated fetal bovine serum (FBS), Dulbecco’s Modified Eagle Medium/Nutrient Mixture F-12 (DMEM/F-12) were purchased from DNA Biotech Co. (Tehran, Iran). Formalin, hydrochloric acid (HCl), and glacial acetic acid were obtained from Dr. Mojallali Industrial Chemical Complex Co. (Tehran, Iran). Figure 1 represents the chemical structure of chitosan, HPMCP, TPP, and BBr used in the preparation of the nanoparticles.

### Cells and animals

Mesenchymal stem cells (MSCs) were obtained from the Pasteur Institute of Iran (Tehran, Iran) and were cultured in low glucose DMEM supplemented with 10% (v/v) FBS. All the cells were cultured at 37 °C in a humidified atmosphere containing 5% CO_2_.

A total of 45 male Wistar rats (200–250 g) were used in this study. The animals were obtained from the Center for Reproduction of Laboratory Animals at Shahroud University of Medical Sciences and were maintained under 12-hour light/12-hour dark cycles and were given water and food ad libitum. All animal experiments were carried out in accordance with the regulations of Shahroud University of Medical Sciences and this study is reported in accordance with ARRIVE guidelines (https://arriveguidelines.org).

### Preparation of nanoparticles

CS/HPMCP and CS/TPP nanoparticles were prepared using the ionic gelation method as previously described [35, 36]. Briefly, 28 mg of CS was dissolved in 10 mL of acetic acid (2%) and the solution was placed on a magnetic stirrer until a transparent solution was obtained (pH 4.2–4.8). Separately, HPMCP (3 mg/mL; dissolved in 0.1 NaOH) and TPP (2 mg/mL; dissolved in deionized water) solutions were prepared and stirred for 30 min (1200 rpm). Moreover, in separate preparations, the HPMCP and TPP solutions were added to the CS solution in a drop-wise manner (at a titration rate of 0.1 mL/minute) and the solutions were stirred (1500 rpm) at room temperature until a volume ratio of 5:2 for both CS:HPMCP and CS:TPP was obtained. In order to prepare nanoparticles with a similar size range, the nanoparticle-containing solutions were stirred for an additional 30 min (1500 rpm).

For the preparation of CS/HPMCP/BBr and CS/TPP/BBr nanoparticle, 28 mg of CS was dissolved in 10 mL of acetic acid (2%) and the solution was placed on a magnetic stirrer until a transparent solution was obtained (pH 4.2–4.8). 28 mg of BBr was added to the CS solution (called CS-BBr solution) and the solution was placed in an ultrasonic bath at 30 ˚C for 30 min. Of note, all of the experiment steps involving BBr were carried out in a dark condition since BBr is a light-sensitive material. The CS-BBr solution was stirred at 30 ˚C for 60 min (1200 rpm) to obtain a transparent solution. Separately, HPMCP (3 mg/mL; dissolved in 0.1 NaOH) and TPP (2 mg/mL; dissolved in deionized water) solutions were prepared and stirred for 30 min (1200 rpm). Moreover, in separate preparations, the HPMCP and TPP solutions were added to the CS-BBr solution in a drop-wise manner (at a titration rate of 0.1 mL/minute) and the solutions were stirred (1500 rpm) at room temperature until a volume ratio of 5:2 for both CS-BBr:HPMCP and CS-BBr:TPP was obtained. In order to prepare nanoparticles with a similar size range, the nanoparticle-containing solutions were stirred for an additional 60 min (1500 rpm).

### Encapsulation efficiency and drug loading capacity

The loading capacity and encapsulation efficiency of CS/HPMCP and CS/TPP nanoparticles were determined by measuring the amount of unentrapped BBr in the supernatant. Briefly, the generated CS/HPMCP and CS/TPP nanoparticles were separated by centrifugation (16,000 rpm, 30 min, and 4 °C) and the amount of free BBr in the supernatant was determined using the Bradford protein assay [37]. The equations used for calculating the loading capacity and encapsulation efficiency of BBr are as follows [38]:

$$\begin{gathered} Encapsulation{\text{ }}\,efficiency{\text{ }}(\% ){\text{ }} = \hfill \\\,\,\,\,\,\,\,\,\,\,\,\,\,\,\,\,\,\,\,\,\,\,\,\,\,\,\,\,\,\,\,\,\frac{{total\,amount\,of{\mkern 1mu} drug\,added - free{\mkern 1mu}\, drug}}{{total\,amount\,of{\mkern 1mu} drug\,added}} \times 100 \hfill \\ \end{gathered}$$

$$\begin{gathered} Loading{\text{ }}capacity{\text{ }}(\% ){\text{ }} = \hfill \\\,\,\,\,\,\,\,\,\,\,\,\,\,\,\,\,\,\,\,\,\,\,\,\,\,\,\,\,\,\,\,\,\,\,\frac{{total\,amount\,of{\mkern 1mu} drug\,added - free{\mkern 1mu}\, drug}}{{weight\,of{\mkern 1mu} nanoparticles}} \times 100 \hfill \\ \end{gathered}$$

### Physicochemical characterization of nanoparticles

#### Particle size, polydispersity index (PI), and zeta potential

The particle size and PI of the freshly prepared CS/HPMCP and CS/TPP nanoparticles were determined by the Dynamic Light Scattering (DLS) method via a particle characterizer device (nanoPartica® SZ-100, Horiba, Japan). Moreover, the same device was also used for the calculation of the zeta potential of the nanoparticles.

#### Fourier transform infrared (FTIR) Spectroscopy

FTIR spectroscopy was carried out to determine any molecular interactions present between the formulation components using the Spectrum GX spectrophotometer (Perkin Elmer, USA). The spectra were collected in the spectral range of 400–4,000 cm^− 1^ with a resolution of 4 cm^− 1^.

#### Morphological analysis using scanning electron microscope (SEM)

The morphology of the synthesized nanoparticles was assessed using SEM (Sigma 300-HV, Zeiss, Germany). Briefly, the synthesized nanoparticles were sputter-coated with gold and then analyzed.

### BBr release from CS/HPMCP/BBr and CS/TPP/BBr nanoparticles in the stimulated gastric fluid (SGF) and stimulated intestinal fluid (SIF) environments

The effect of pH on the release ability of BBr from CS/HPMCP and CS/TPP nanoparticles in SGF (pH 1.2) and SIF (pH 7.4) was assessed in eight different time points (15, 30, 60, and 90 min, and 2, 3, 4, 6, and 8 h) [39]. Briefly, 2 mL of CS/HPMCP/BBr and 2 mL of CS/TPP/BBr nanosuspensions were separately placed in dialysis tubing (12 kDa) and then they were placed in 48 mL of SGF and SIF at 37 °C and with gentle shaking (50 rpm). At appropriate intervals, 2 mL of SGF and SIF was taken and replaced by fresh medium. The quantity of the released BBr in the taken SGF and SIG samples was assessed using Ultraviolet–visible (UV-VIS) spectroscopy at a maximum wavelength (λ_max_) according to the standard curve of BBr [40]. Of note, to avoid light-induced BBr decomposition, all of the mentioned steps were performed in the dark.

### Cell viability assay

MTT assay was used to investigate the possible cytotoxic effects of different concentrations of CS/HPMCP, CS/TPP, CS/HPMCP/BBr, and CS/TPP/BBr nanoparticles on the viability of MSCs. Briefly, 1 × 10^4^ MSCs were seeded in different wells of a 96-well cell culture plate and then they were incubated at 37 ºC for 24 h. Next, the cells were treated with different concentrations of each of the indicated nanoparticles (1, 0.5, 0.25, 0.125, 0.0625, 0.03125, and 0.015625 mg/mL) for 24 and 72 h. Next, 10 µL of MTT solution (5 mg/mL) was added to each well which was followed by a 4-hour incubation. Afterward, the media was gently aspirated and 100 µL of pure DMSO was added to each well and the absorbance was read at 570 and 690 nm using an ELISA reader [41] (Cytation 5 BioTek, USA).

### In vivo assays

The animals were randomly categorized into nine experimental groups (n = 5) including [1] control group (normal rats with no liver damage; named “control”), [2] sham group (alcohol-receiving animals with no previous treatment; named “EtOH”), [3] alcohol-receiving, previously treated with CS/HPMCP/BBr nanoparticles (named “CS/HPMCP/BBr”), [4] alcohol-receiving, previously treated with CS/HPMCP nanoparticles (named “CS/HPMCP”), [5] alcohol-receiving, previously treated with CS/TPP/BBr nanoparticles (named “CS/TPP/BBr”), [6] alcohol-receiving, previously treated with CS/TPP nanoparticles (named “CS/TPP”), [7] alcohol-receiving, previously treated with free CS (named “CS”), [8] alcohol-receiving, previously treated with BBr (named “BBr”), and [9] alcohol-receiving, previously treated with free CS and BBr (named “CS/BBr”).

For 21 days, CS/HPMCP/BBr, CS/HPMCP, CS/TPP/BBr, CS/TPP, CS, BBr, and “CS/BBr” suspensions (20 mg/kg) were given to the rats in the treated groups through the gastric gavage route. Two hours after this, 45% ethanol was given to the treatment groups (20 mg/kg) through gastric gavage. 24 h after performing the last treatment, the animals were anesthetized with an intraperitoneal injection (75–100 mg/kg of 10% ketamine and 10 mg/kg of xylazine 2%). Blood samples were quickly collected from the heart of the animals and the sera were obtained through centrifugation (2000 × g for 10 min at 4 °C using a refrigerated centrifuge). The obtained sera were stored at -80 ºC for further analysis. Moreover, the livers of the animal models were excised and gently washed using physiological serum and placed in 40% formaldehyde solution and kept for further assessments.

#### Biochemical assessments

The serum levels of aspartate aminotransferase (AST), alanine aminotransferase (ALT), alkaline phosphatase (ALP), and gamma-glutamyl transpeptidase (GGT) were determined using commercially available kits (ParsAzmoon, Tehran, Iran) according to manufacturer’s instructions. Moreover, liver functionality was further studied by determining the relative concentration of MDA and glutathione peroxidase (GPx) using the lipid peroxidation MDA assay and GPx assay, respectively.

#### Histopathological assays

Hematoxylin and eosin (H&E) and Masson’s trichrome staining were used for the histopathological evaluation of the liver of the animals. In brief, livers were fixed using 10% formalin and were embedded in paraffin. 5 μm slices were prepared from the fixed tissues and were stained with H&E and Masson’s trichrome staining, separately. The prepared slides were analyzed under a light microscope.

### Statistical analysis

One-way and two-way analysis of variance (ANOVA) was performed for statistical analyses using Graph Pad Prism 9 (GraphPad Software, USA). A *p*-value < 0.05 was considered statistically significant.

## Results

### Mean particle size, PI, and zeta potential of the prepared nanoparticles

**Fig. 2:**
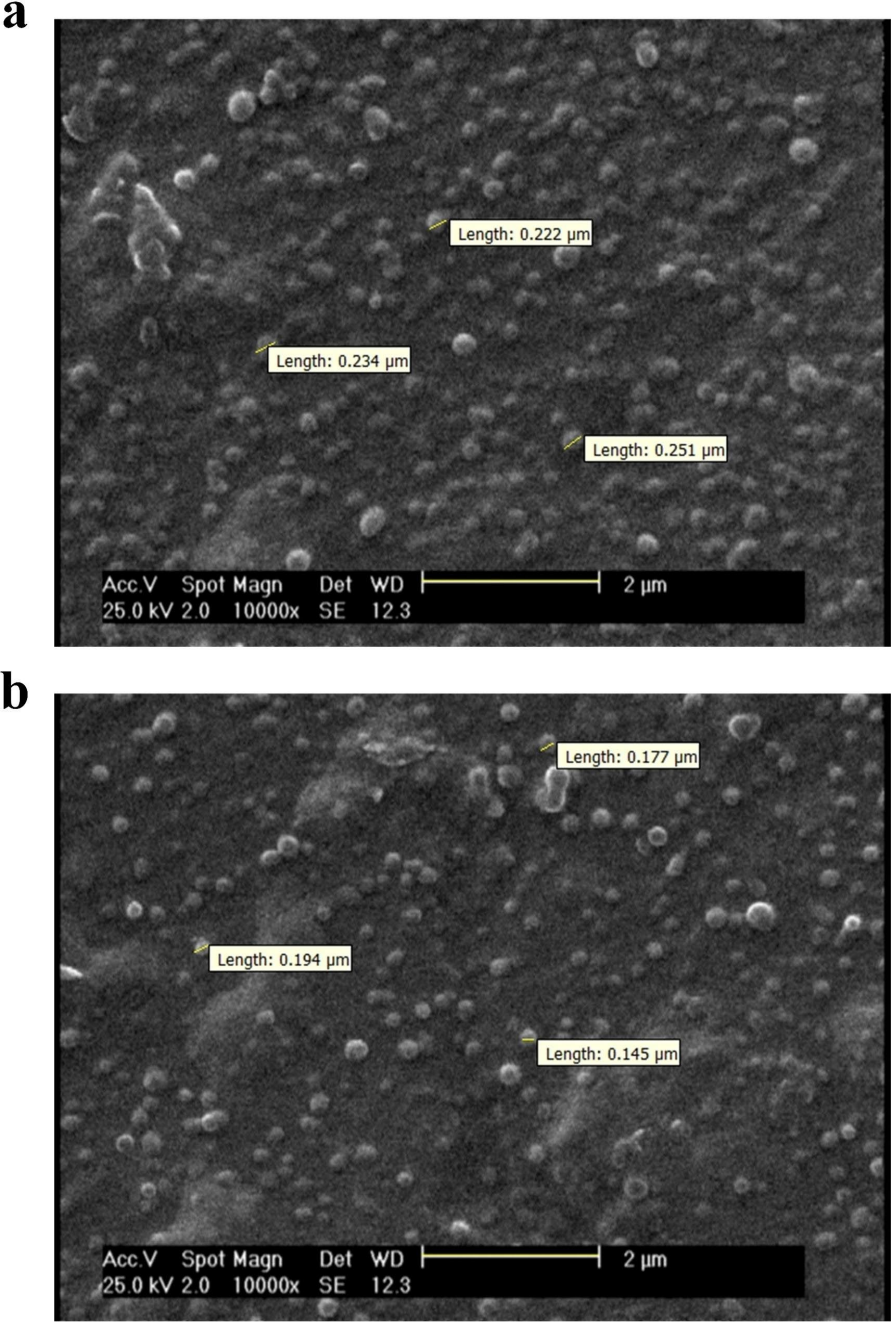
SEM images of CS/HPMCP/BB and CS/TPP/BBr nanoparticles demonstrate the morphology and the average size of the prepared nanoparticles. **a**: CS/HPMCP/BBr nanoparticles. **b**: CS/TPP/BBr nanoparticles

The average size of the prepared nanoparticles before and after BBr loading was determined using DLS. In detail, CS/HPMCP/BBr and CS/TPP/BBr nanoparticles had an average size of 260 ± 23 and 198 ± 17 nm, respectively. Moreover, CS/HPMCP nanoparticles exhibited an average size of 245 ± 42 nm and CS/TPP nanoparticles had an average size of 172 ± 21 nm. According to SEM images presented in Fig. 2a and b, CS/HPMCP/BBr nanoparticles had a particle size ranging from 222 to 251 nm, with an average size of 235.5 nm. On the other hand, CS/TPP/BBr nanoparticles had a smaller particle size ranging from 145 to 194 nm, with an average size of 172 nm. In regard to the PI, CS/HPMCP/BBr and CS/HPMCP nanoparticles had a PI of 0.28 ± 0.024 and 0.25 ± 0.2, respectively. Moreover, CS/TPP/BBr and CS/TPP nanoparticles demonstrated a PI of 0.27 ± 0.02 and 0.27 ± 0.1, respectively. The zeta potential of CS/HPMCP/BBr nanoparticles was about 32 ± 0.25 mV (27 ± 0.35 mV for CS/HPMCP nanoparticles), whereas CS/TPP/BBr nanoparticles had a zeta potential of about 28 ± 0.31 mV (25 ± 0.41 mV for CS/TPP nanoparticles).

### FTIR

**Fig. 3:**
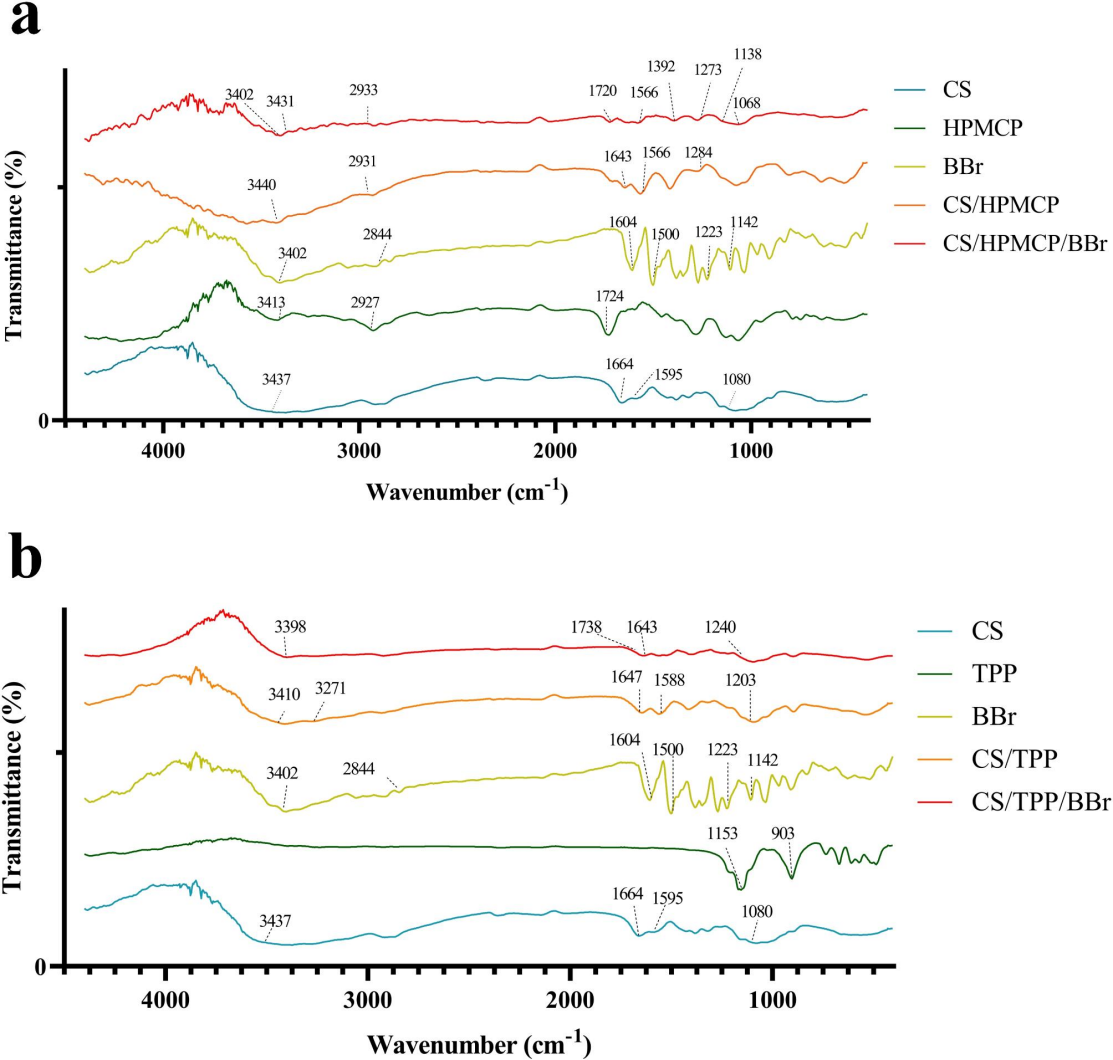
FTIR spectra of the materials used in the preparation of investigated CS/HPMCP/BB and CS/TPP/BBr nanoparticles. **a**: spectra of CS, HPMCP, BBr, as well as CS/HPMCP and CS/HPMCP/BBr nanoparticles. **b**: spectra of CS, TPP, BBr, as well as CS/TPP and CS/TPP/BBr nanoparticles

The FTIR spectra of CS, HPMCP, TPP, BBr, as well as CS/HPMCP, CS/HPMCP/BBr, CS/TPP, and CS/TPP/BBr nanoparticles are presented in Fig. 3a and b. According to the results, CS has two strong peaks in 1596 cm^− 1^ and 1664 cm^− 1^ which has been attributed to CONH_2_ and NH_2_ groups. The fluctuation in these peaks in the spectrum of CS/HPMCP nanoparticles in comparison with free CS is a sign of interaction between the NH_3_ group of CS and the COO^−^ group of HPMCP. This interaction is recognized through the strong decline of the amid band in 1655 cm^− 1^. The broad peak in 3400 cm^− 1^ has been attributed to the stretching vibration of the NH_2_ and OH groups. Such peaks are more visible and robust in the CS group which is an indicator of strong hydrogen bonds. Also, this peak can be attributed to CH_2_ interactions in the CS group. The elevation seen from 1203 cm^− 1^ to 1240 cm^− 1^ in CS/TPP nanoparticles in comparison with the free CS group is an indicator of P-O interactions. Moreover, the fluctuation of the peaks from 1647 cm^− 1^ to 1738 cm^− 1^ and from 1588 cm^− 1^ to 1643 cm^− 1^ in CS/TPP nanoparticles in comparison with CS are due to C-O and N-H interactions, respectively. Moreover, BBr has sharp peaks in 1500 cm^− 1^ to 3000 cm^− 1^ regions. BBr is bonded to CS through forming amid bonds with carboxyl groups or by forming hydrogen bonds with carbonyl groups (179). The FI-IR spectra of BBr, CS/HPMCP/BBr, and CS/TPP/BBr clearly demonstrate that CS and BBr are bonded to each other without any structural changes.

### Loading capacity and encapsulation efficiency

In this experiment, the encapsulation efficiency was calculated for both CS/HPMCP and CS/TPP nanoparticles and it was reported to be 75.79% and 80.05%, respectively. Moreover, loading capacity of CS/HPMCP and CS/TPP nanoparticles was calculated as 79.78% and 84.26%, respectively.

### In vitro assays

#### Drug release assay

**Fig. 4:**
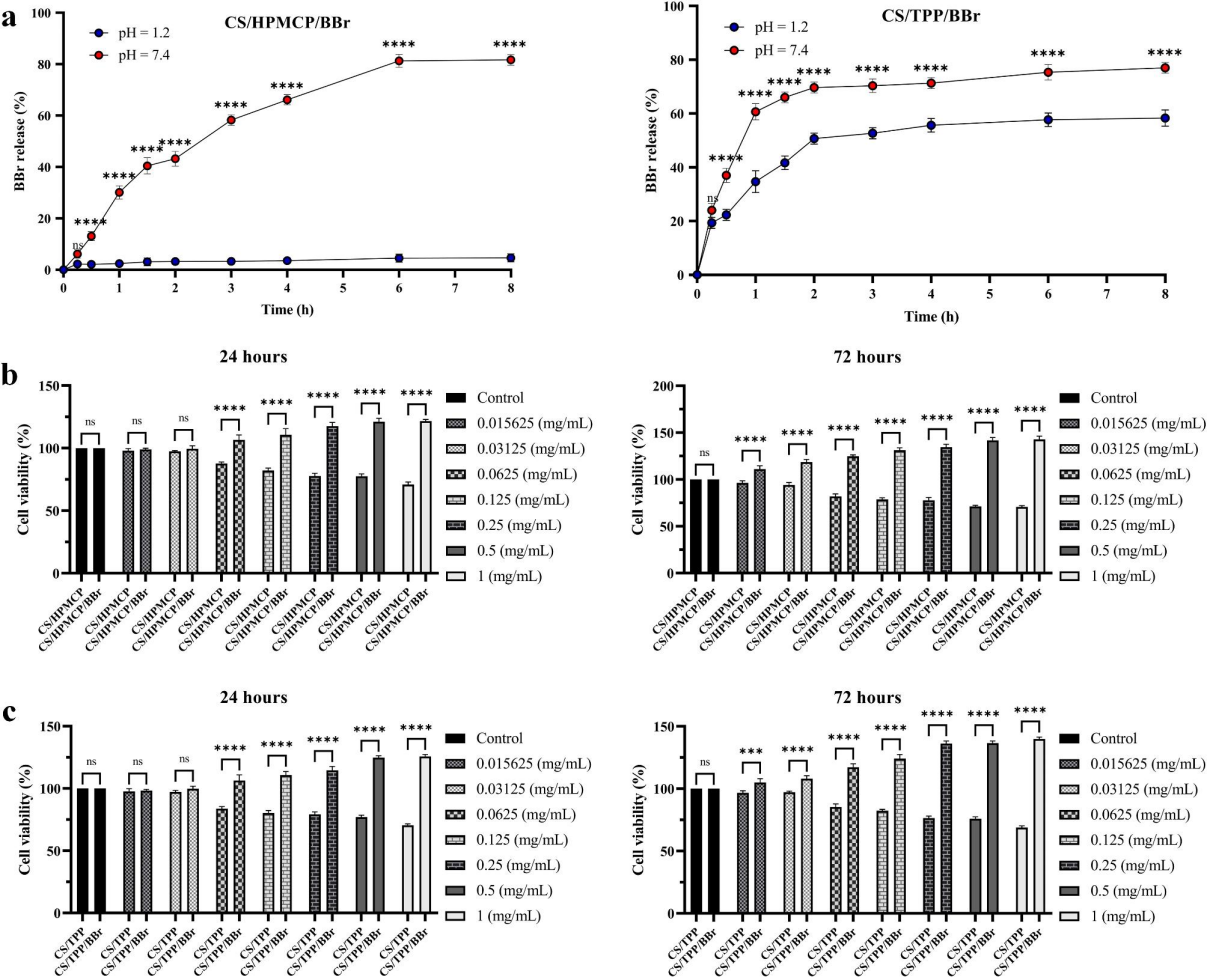
The release graph and cell viability assay results of CS/HPMCP/BBr and CS/TPP/BBr nanoparticles. **a**: the release percentage of BBr from CS/HPMCP/BBr (left panel) and CS/TPP/BBr (right panel) nanoparticles over the course of 8 h in the SGF (pH = 1.2) and SIF (pH = 7.4) environments. BBr release percentage of CS/HPMCP/BBr and CS/TPP/BBr nanoparticles was significantly higher in SIF settings than those of CS/HPMCP/BBr and CS/TPP/BBr in SGF settings, respectively at all of the investigated time intervals except for the first 15 min (*p* < 0.0001). All experiments were carried out in triplicate (*n* = 3). **b**: the effects of the exposure of CS/HPMCP/BBr and CS/HPMCP nanoparticles on the viability of MSCs after 24 h (left panel) and 72 h (right panel). After 24 h, CS/HPMCP/BBr nanoparticle exposure resulted in significantly higher cell viability rates in comparison with CS/HPMCP at 0.0625, 0.125, 0.25, 0.5, 1 mg/mL concentrations (*p* < 0.0001 for all of the significant groups). After 72 h, CS/HPMCP/BBr nanoparticle exposure resulted in significantly higher cell viability rates in comparison with CS/HPMCP at all of the investigated concentrations (*p* < 0.0001 for all of the significant groups). **c**: the effects of the exposure of CS/TPP/BBr and CS/TPP nanoparticles on the viability of MSCs after 24 h (left panel) and 72 h (right panel). After 24 h, CS/TPP/BBr nanoparticle exposure resulted in significantly higher cell viability rates in comparison with CS/TPP at 0.0625, 0.125, 0.25, 0.5, and 1 mg/mL concentrations (*p* < 0.0001 for all of the significant groups). After 72 h, CS/TPP/BBr nanoparticle exposure resulted in significantly higher cell viability rates in comparison with CS/TPP at all of the investigated concentrations (*p* < 0.0001 for 0.015625 mg/mL concentration group and *p* < 0.0001 for the other groups). “ns” for statistically non-significant, *** for *p* < 0.001, and **** for *p* < 0.0001. All experiments were carried out in triplicate (*n* = 3)

In this experiment, we assessed the effect of pH on the release ability of CS/HPMCP/BBr and CS/TPP/BBr nanoparticles for releasing BBr in in vitro settings of SGF (pH 1.2) and SIF (pH 7.4) at eight different time points (15, 30, 60, and 90 min, and 2, 3, 4, 6, and 8 h after the starting time of the experiment) [39]. According to the results presented in Fig. 4a, the pH of the environment was a determining factor in the drug-releasing rate of the nanoparticles. In detail, CS/HPMCP/BBr nanoparticles demonstrated a BBr release percentage of no more than 4.6% in the SGF environment; however, this pattern was remarkably different in the SIF environment as CS/HPMCP/BBr nanoparticles demonstrated a BBr release rate of 43.2% after the first 2 h reaching to 81.6% after eight hours since the start of the experiment. Statistical analyses indicated that the BBr release percentage of CS/HPMCP/BBr nanoparticles was significantly higher (*p* < 0.0001) in the SIF environment in comparison with the SGF environment at all of the investigated time intervals except for the first 15 min.

In regard to CS/TPP/BBr nanoparticles, the releasing pattern was different in the SGF environment. In detail, CS/TPP/BBr nanoparticles managed to release 50.6% of the BBr in the first 2 h reaching 58.3% after eight hours since the start of the experiment. In the SIF environment, CS/TPP/BBr nanoparticles demonstrated a releasing rate of 69.6% in the first 2 h and a releasing rate of 77% after 8 h. In a similar fashion to CS/HPMCP/BBr nanoparticles, CS/TPP/BBr nanoparticles also exhibited significantly higher (*p* < 0.0001) release rates in the SIF environment in comparison with the SGF environment at all of the investigated time intervals except for the first 15 min. Such results indicate even though CS/TPP/BBr nanoparticles demonstrated a high release rate in both of the simulated conditions, it can be concluded that both of the nanoparticles can significantly manage the release of their cargo in the gastrointestinal tract.

#### Cell viability assay

In this experiment, the MTT assay was used to determine the effects of BBr delivery using different concentrations of CS/HPMCP/BBr and CS/TPP/BBr nanoparticles on the viability of MSCs. Of note, in the case of CS/HPCMP/BBr and CS/TPP/BBr nanoparticles, according to the results of the loading capacity and encapsulation efficiency, around 80% of the prepared nanoparticles were loaded with BBr. Therefore, around 80% of each of the indicated concentrations of CS/HPCMP/BBr and CS/TPP/BBr nanoparticles were drug-loaded. In the case of CS/HPMCP and CS/HPMCP/BBr nanoparticles (Fig. 4b), CS/HPMCP/BBr nanoparticles significantly elevated the cell viability level in all of the experimented concentrations in comparison with CS/HPMCP nanoparticles in 72 h (*p*-value < 0.0001 for all of the groups). The same pattern was also observed after 24 h with the exception that two of the lowest investigated concentrations of CS/HPMCP/BBr nanoparticles (0.015625 and 0.03125 mg/mL) did not significantly elevate the cell viability level in comparison with CS/HPMCP nanoparticles.

Additionally, CS/TPP and CS/TPP/BBr nanoparticles demonstrated very similar behavior in comparison with CS/HPMCP and CS/HPMCP/BBr nanoparticles, respectively (Fig. 4c). In detail, after 24 h, the concentration of 0.0625 mg/mL and higher concentrations of CS/TPP/BBr significantly increased cell viability rate in comparison with CS/TPP nanoparticles (*p*-value < 0.0001 for all of the groups). Moreover, after 72 h, treatment with all of the concentrations of CS/TPP/BBr nanoparticles significantly increased the cell viability rate of MSCs in comparison with CS/TPP nanoparticles (*p*-value < 0.0001 for all of the groups). Such data indicate that long-term exposure of human MSCs to CS/HPMCP/BBr and CS/TPP/BBr nanoparticles does not negatively affect normal cellular functioning and viability of the cells.

### In vivo experiments

#### Enzymatic analysis

**Fig. 5:**
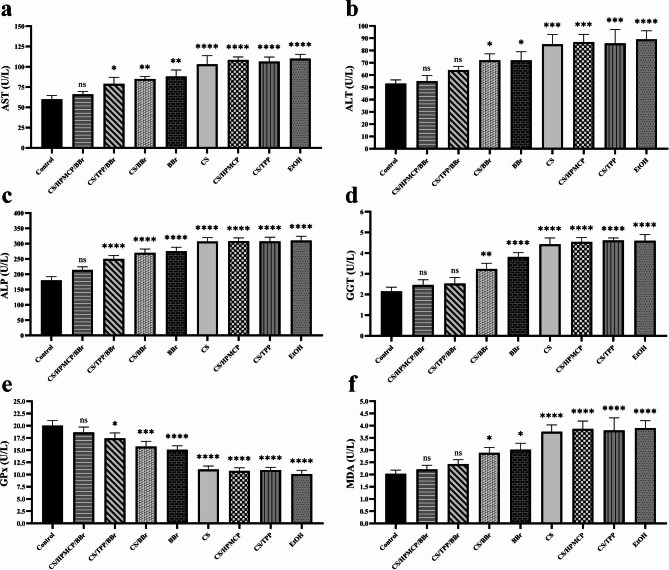
The plasma level of AST, ALT, ALP, GGT, GPx, and MDA in rat models of alcohol-induced liver damage in different experimental groups. **a**: the plasma level of AST. **b**: the plasma level of ALT. **c**: the plasma level of ALP. **d**: the plasma level of GGT. **e**: the plasma level of GPx. **f**: the plasma level of MDA. Experimental groups are statistically compared with the control group. “ns” indicates statistically non-significant, * for *p* < 0.05, ** for *p* < 0.01, *** for *p* < 0.001, and **** for *p* < 0.0001. Data are presented as means ± SD (*n* = 3)

To investigate the protective effects of CS/HPMCP/BBr and CS/TPP/BBr nanoparticles against alcohol-induced hepatotoxicity, the level of different hepatic enzymes was evaluated in each of the experimental groups. According to our observations, alcohol delivery resulted in significant deviance in the level of hepatic enzymes including AST, ALT, ALP, GGT, GPx, and MDA. In detail, the serum levels of AST, ALT, ALP, GGT, and MDA in the EtOH group were significantly higher in comparison with the control group (*p* < 0.0001 for all of the enzymes). Moreover, the level of GPx in the EtOH group was significantly lower than the control group (*p* < 0.0001; Fig. 5). These findings indicate that alcohol delivery results in remarkable liver damage and consequently impaired liver functioning. In regards to the experimental performance of the tested materials, only CS/HPMCP/BBr treatment prevented significant deviance in the level of all of the tested liver enzymes in comparison with the control group, after the animals were given alcohol. Moreover, CS/TPP/BBr treatment only prevented significant deviance in the level of ALT, GGT, and MDA in comparison with the control group after the animals were given alcohol, indicating a partial response in comparison with its counterpart CS/HPMCP/BBr.

#### Histopathological analysis

**Fig. 6:**
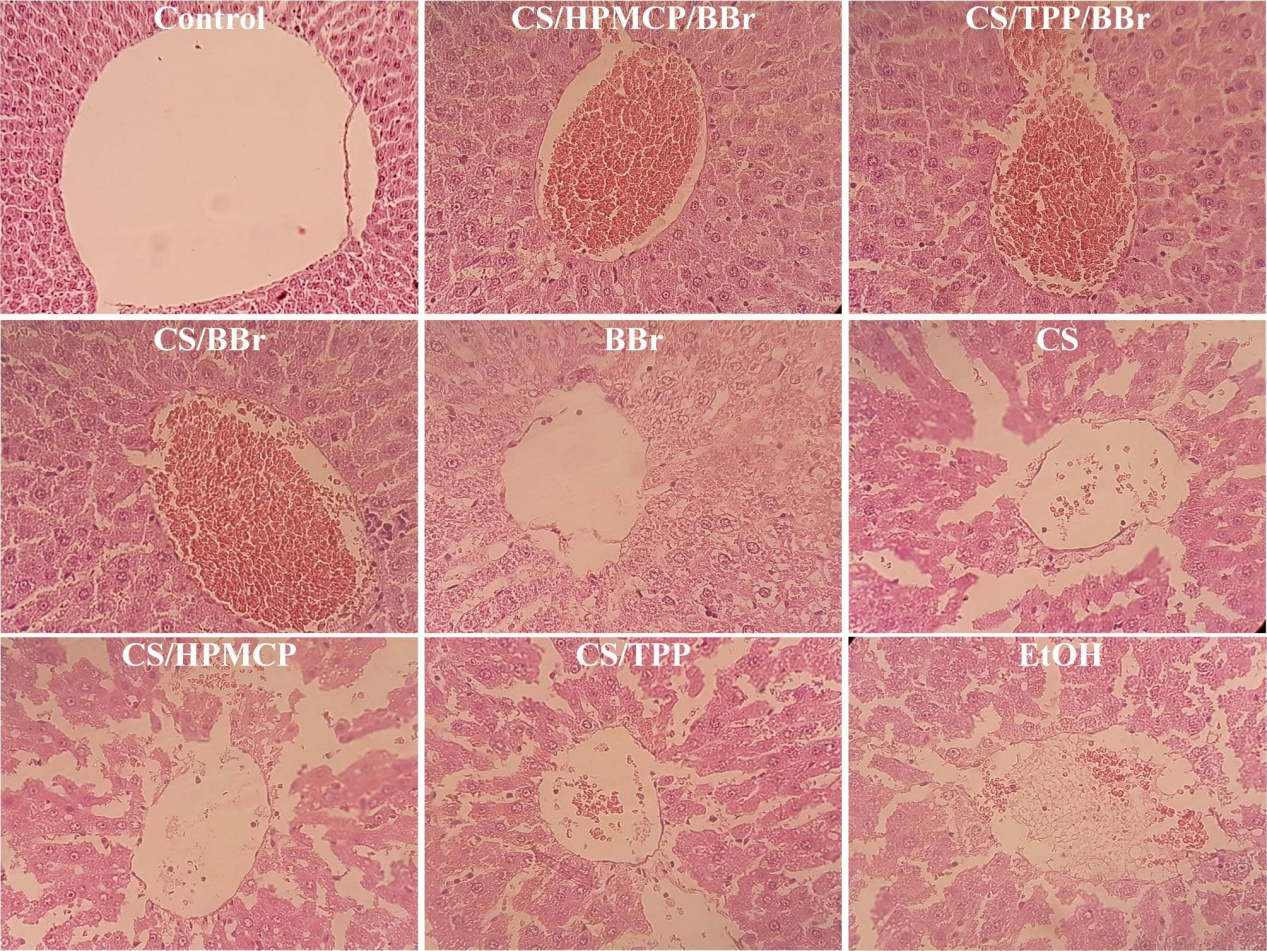
H&E staining of the liver of alcohol-induced liver damage rat models. The magnification of the images is ×40

Microscopic evaluation of the liver tissues via H&E staining in the control group demonstrated that the liver lobules have a clear structure with the hepatic cords arranged from the central veins to the periphery. However, in the EtOH group, the findings indicated the abnormal lobular structure of the liver, the irregular structure of the liver cord, and the interstitial infiltration of inflammatory cells. In the CS group, the destruction and disorganization of the liver cord, and infiltration of inflammatory cells were observed similar to the EtOH group. The same results were observed in the CS/HPMCP and CS/TPP groups. Therefore, it is safe to conclude that CS, HPMCP, or TPP do not solely demonstrate any remarkable protective effects. In the CS/HPMCP/BBr group, the penetration of inflammatory cells, the destruction of the liver cells, and the disruption of the hepatic cord were remarkably prevented. In the CS/TPP/BBr group, relatively more tissue damage was observed in comparison with the CS/HPMCP/BBr group. Additionally, in the CS/BBr group as well as the BBr group, the disorganization in the liver cord, infiltration of inflammatory cells, and destruction of liver cells were observed (Fig. 6).

**Fig. 7:**
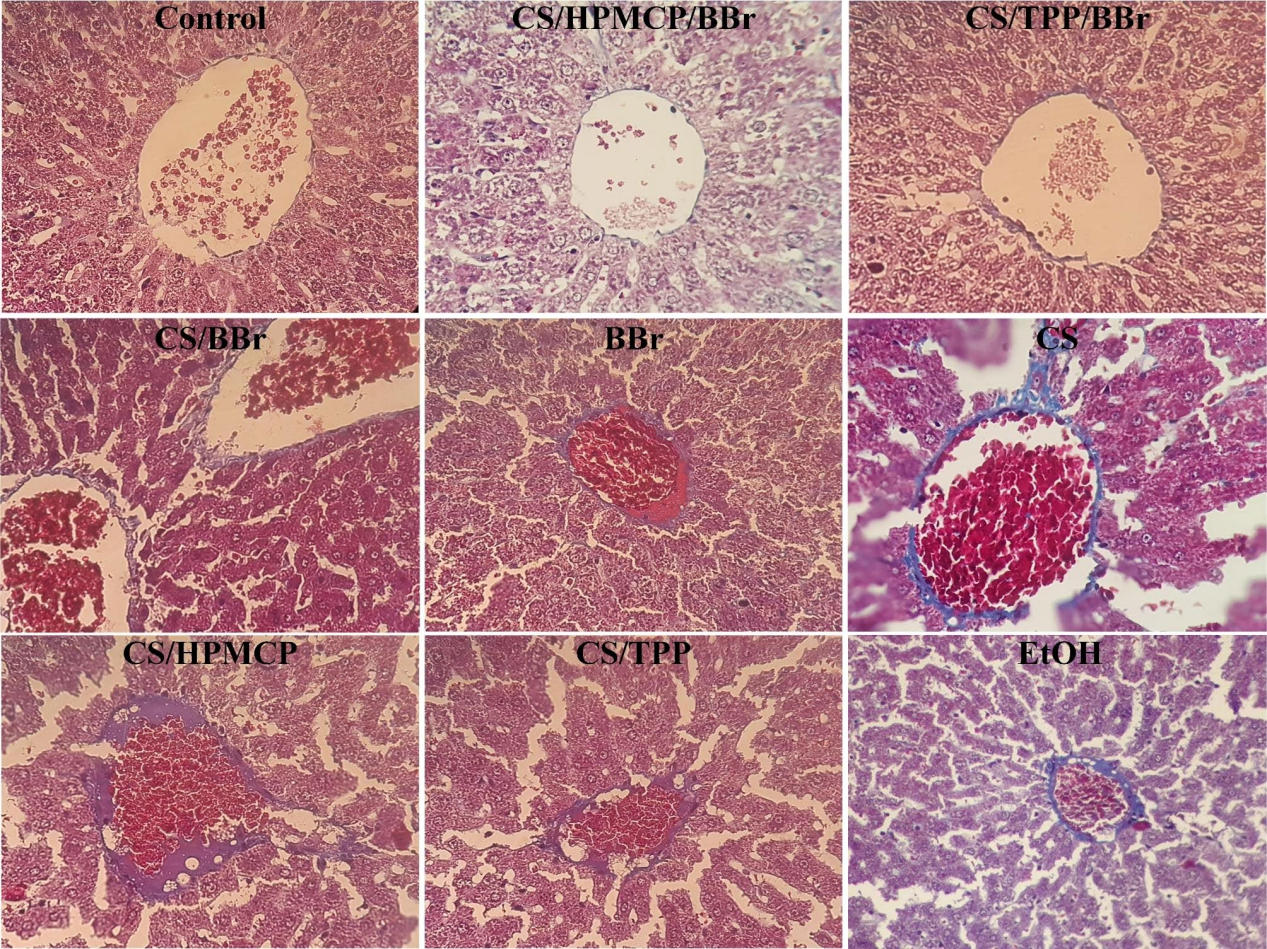
Masson’s trichrome staining of the liver of alcohol-induced liver damage rat models. The magnification of the images is ×40

Additionally, the protective effects of the nanoparticles were investigated using TCM staining (Fig. 7). In detail, in the liver tissue samples of the control group, no collagen fibers were seen around the central vein and the cells and the liver cord had a preserved structure. On the other hand, in the EtOH group, abnormal liver lobular structure, irregular structure of the liver cord, and abundant collagen fibers around the central vein were documented. Similarly, in the CS, CS/HPMCP, and CS/TPP groups, the destruction and irregularity of the liver cord, the infiltration of inflammatory cells, and numerous collagen strands were present; suggesting no remarkable protective effects for CS, HPMCP, and TPP as single agents and supporting what was indicated in the H&E staining assay results. In the CS/HPMCP/BBr group, the disorder in the liver cord was significantly reduced, the endothelium cells were placed side by side, and the collagen layers around the central vein were not present. Similar results were also observed in the CS/TPP/BBr group; however, the destruction and damage were still observed and collagen fibers were present. Also, in both the CS/BBr and BBr groups, the destruction of the liver cells and the collagen fibers, and more disarray in the liver cord were observed.

## Discussion

Chronic alcohol consumption is a globally major public health challenge and is known as one of the main factors for non-communicable diseases [42]. Since alcohol is mainly metabolized in the liver, this organ is considered one of the main sites of damage after alcohol consumption [43]. Liver diseases caused by alcohol consumption include steatosis, steatohepatitis, cirrhosis, and hepatocellular carcinoma [43]. Ethanol metabolism in the liver causes an increase in the level of ROS, which leads to an imbalance of oxidation and reduction. In this regard, antioxidants could be considered a solution for restoring the balance of oxidation and reduction [44–46]. Medicinal antioxidants could have beneficial effects in reducing the occurrence of ethanol-induced changes in cellular lipids, proteins, and nucleic acids, and can act as a natural antioxidant defense booster by trapping free radicals, causing an interruption in the peroxidation process [45, 47–49]. BBr is a yellow alkaloid present in numerous plants including barberry [50, 51]. Accumulating evidence suggests that BBr has numerous properties including anti-inflammatory, antioxidant, anti-convulsant, anti-depressant, anti-Alzheimer, anti-cancer, anti-arrhythmic, anti-viral, anti-bacterial, and anti-diabetic properties [52–54]. Also, BBr can reduce the toxicity of chemical toxins in the brain, heart, kidney, liver, and lung through its antioxidant, anti-inflammatory, and anti-apoptotic properties, and modulation of the mitogen-activated protein kinase (MAPK) and nuclear factor κB and signaling pathways. (NF-κB) [14, 55, 56]. However, the low level of BBr bioavailability, absorption, and solubility are the known major obstacles to its systemic administration. In fact, only 0.5% of ingested BBr is absorbed in the small intestine and this amount decreases to 0.35% by the time it enters the bloodstream. In this regard, it is believed that nano-based formulations are ideal candidates to increase the absorption rate of BBr since nano-scale compounds can be absorbed in the intestine with the desired speed and concentration [57]. CS nanoparticles have interesting biological properties such as non-toxicity, biocompatibility, biodegradability, mucosal adhesion, and the ability to penetrate through the epithelial tight junctions; therefore, they are considered suitable carriers for drug delivery, including oral drug delivery [32, 58, 59].

It should also be stated that CS nanoparticles are easily decomposed and dissolved in acidic conditions of the stomach. CS nanoparticles formulated with HPMCP (as a pH-sensitive polymer) have good film-forming properties, rapid dissolution at intestinal pH, and stable physical and chemical properties. Hydroxypropyl methylcellulose (HPMC) acts as the backbone and is esterified by phthalic anhydride. It is relatively insoluble in water and the stomach, and it can expand and dissolve quickly in the upper part of the intestine. This polymer provides superior acid stability and demonstrates enhanced adhesion and intestinal penetration capacity compared to the CS/TPP form [34]. The pH-sensitive HPMCP polymer is used as a cross-linker to stabilize CS nanoparticles in the acidic conditions of the stomach and to enable them to release their cargo in the intestinal environment with a pH of ≥ 5.5. HPMCP is a pH-sensitive polymer (the critical pH of decomposition can be controlled by the content of phthalates) that protects any given drug loaded into nanoparticles in the acidic conditions of the stomach and releases them in the intestine. One of the ways to reduce the side effects in the digestive system is to use polymers such as HPMCP since such polymers can be advantageous as they can induce the release of the loaded drugs at the absorption site [34]. The aim of this study was to compare the protective effects of CS/HPMCP nanoparticles loaded with BBr and CS/TPP loaded with BBr to study the cargo-releasing ability of the indicated nanoparticles in the intestine. This is a highly important research topic in regard to the challenge of excessive alcohol use and hepatotoxic diseases.

In this study, CS/HPMCP/BBr and CS/TPP/BBr nanoparticles were successfully prepared using the ionic gelation technique [60]. To prepare stable nanoparticles, the controllable conditions in the synthesis and the related formulation parameters were optimized. pH-sensitive polymers in the structure of nanoparticles can be used to prevent the premature release of drugs or active substances from nanoparticles in the upper gastrointestinal tract. This strategy prevents the reduction of drug concentration in the intestine and subsequently prevents the reduction of the absorption and effectiveness of the drug. In the current study, CS nanoparticles were cross-linked using two different cross-linkers. The use of HPMCP in the synthesis of CS nanoparticles reduces the leakage of BBr in the upper part of the digestive tract and enables the specific delivery of BBr to the intestine. HPMCP, as a non-toxic pH-sensitive polymer with biodegradability and a good biocompatibility profile, has recently been used as a drug coating material for enteral drug delivery [61–63]. This polymer is insoluble in the gastric pH, while it is completely soluble in the intestinal pH [63, 64]. Hence, HPMCP was used as an enteric-coated substance to resist gastric acid [63, 64]. In this method, drug dissolving starts in the small intestine and the maximum absorption of the drug occurs in the intestine. Studies on the release of BBr in the simulated environments of the stomach (pH 1.2) and the intestine (pH 7.4) have demonstrated that pH had a great effect on the release of BBr from CS/HPMCP/BBr and CS/TPP/BBr nanoparticles. Moreover, the release rate of BBr from CS/HPCMP/BBr nanoparticles at pH 7.4 was much higher than that of pH 1.2, and effective release was observed in the simulated environment of the stomach (pH = 1.2). On the other hand, the release of BBr from CS/TPP/BBr nanoparticles in the SIF environment was slightly higher than its release in the SGF environment; however, the difference was still significant. These observations demonstrated the inhibition of drug release in the acidic pH of the stomach, as drugs do not effectively release until the nanoparticles are in the intestine. Also, a slow and continuous release of the drug from CS/TPP/BBr nanoparticles in the intestine was observed. Investigation of cytotoxicity effects using the MTT assay demonstrated increased survival and proliferation of MSCs during treatment with various concentrations of CS/HPMCP/BBr and CS/TPP/BBr nanoparticles. MSCs were used as the target cell line for cellular assessments of this experiment since we did not have access to a normal nonmalignant liver cell line. This factor can be considered one of the shortcomings of this study which requires further in-depth investigations.

Furthermore, the administration of CS/HPMCP/BBr nanoparticles had significant protective and preventive effects on hepatotoxicity caused by ethanol administration in rats. This effect was confirmed by the improvement of macroscopic and histological damage and the normal serum level of AST, ALT, ALP, GGT, GPx, and MDA. Our results demonstrated the beneficial effects of CS/HPMCP/BBr and CS/TPP/BBr nanoparticles in preventing hepatotoxicity in rats in vivo. 21 days of prevention through the oral administration of CS/HPMCP/BBr or CS/TPP/BBr nanoparticles mediated remarkable protective effects against hepatotoxicity, which was determined by macroscopic, enzymatic, and histological examinations. Moreover, in this study, the level of antioxidant enzymes (MDA & GPx) in the animals treated with CS/HPMCP/BBr nanoparticles had non-significant change as compared to the control group, which can indicate the remarkable protective effects of CS/HPMCP/BBr nanoparticles. Lipid peroxidation is the basic cellular damage process caused by oxidative stress and is considered a hallmark of oxidative stress in which ROS interact with unsaturated fatty acids leading to the formation of lipid products such as MDA that can damage cellular membrane components and cause necrosis and inflammation [65–67].

The present study demonstrated that CS/HPMCP/BBr nanoparticles can mediate higher protective effects than CS/TPP/BBr nanoparticles. Also, CS/HPMCP/BBr and CS/TPP/BBr nanoparticles demonstrated superior protective effects in comparison with the bulk form of BBr. Our results are similar to the results reported by Li et al. which have reported the hepatoprotective properties of BBr based on antioxidant enzymes [68]. Moreover, Makhlof et al. have also reported the slow and controlled drug release loaded in CS/hydroxypropyl nanoparticles. These researchers reported the stability of methyl cellulose phthalate in the intestinal pH and gastric pH [34]. According to the results of our study and other similar reports, CS/HPMCP/BBr nanoparticles can be described as suitable and affordable products with an easy production method as a protective factor against alcoholic hepatotoxicity caused by continuous ethanol consumption. It is worth mentioning that more in vivo assessments can further support the protective effects reported in this study and also elucidate the possible shortcomings and limitations of this method.

## Conclusion

In this study, CS/HPMCP/BBr and CS/TPP/BBr nanoparticles were prepared. The ideal biocompatibility of these nanoparticles, their low toxicity, sensitivity to pH, and proper drug release characteristics make them suitable carriers for oral drug delivery as a protective agent against hepatotoxicity caused by ethanol consumption. Due to the favorable drug release capacity of CS/HPMCP/BBr nanoparticles in comparison with CS/TPP/BBr nanoparticles at the indicated target sites, CS/HPMCP nanoparticles are considered more effective carriers for therapeutic substances, such as BBr, with the aim of mediating protective effects against hepatotoxicity caused by ethanol consumption. This study can also serve as a pipeline for the preparation of nanoparticles ideal for the delivery of various types of cargo which may be damaged upon oral administration/consumption in the low pH of the stomach but are intended to be absorbed in the intestine.

## Data Availability

The datasets used and/or analyzed during the current study available from the corresponding author on reasonable request.
